# Using animation to teach breastfeeding physiology: a proof of concept study

**DOI:** 10.1186/s13006-021-00368-2

**Published:** 2021-02-18

**Authors:** Nicki Hartney, Dolores Dooley, Cate Nagle

**Affiliations:** 1 School of Nursing and Midwifery, Faculty of Health, Deakin University, Geelong Waterfront Campus, Locked Bag 20000, Geelong, VIC 3220 Australia; 2 Centre for Nursing and Midwifery Research, James Cook University, Townsville, QLD Australia; 3 Townsville Hospital and Health Service, Townsville, QLD Australia

**Keywords:** Breastfeeding, Breastmilk, Midwifery, Midwifery students, Animation, Midwifery education, Instructional design, Professional development

## Abstract

**Background:**

Breastfeeding provides the optimal nourishment for infant and child health and supporting mothers to breastfeed is a global health priority. Midwives are uniquely placed to provide breastfeeding education and support to the woman and it is imperative that they have a sound understanding of the physiological underpinnings of breastfeeding. However, midwifery students and some midwives continue to struggle with the complex physiology of lactation. The purpose of this study was to evaluate an instructional animation resource to teach breastfeeding physiology to student and practicing midwives. Further, this study also offers insights into how student and practicing midwives accept novel approaches to learning.

**Methods:**

A cross-sectional survey design using both quantitative and qualitative approaches was employed in this proof of concept study. The setting was online with midwifery students recruited from Deakin University and registered midwives recruited from the Australian College of Midwives membership. Snowball sampling was also employed to recruit midwives through professional networks of the research team. The quantitative part of this study included a structured online questionnaire for midwives and midwifery students and descriptive statistics were used to present the quantitative data. The qualitative data were collected from open-ended questions on the questionnaire and a deductive approach was used for analysing the data.

**Results:**

This proof of concept study collected data from 110 participants and provides evidence for the use of animation as an effective pedagogical tool to explain complex concepts. The animated instructional resource was viewed favourably by both the midwifery students and practicing midwives.

**Conclusions:**

The findings from this study, support the pedagogical advantages of animated instructional resources for teaching complex physiology. Further, educators should be encouraged and feel confident to develop and use animation technology as both an engaging and effective teaching resource especially for complex concepts.

## Background

The short and long-term health benefits of breastfeeding for both the mother and child are well established [1, 2] and the World Health Organization recommend that infants be exclusively breastfed for the first 6 months of life [3]. However, despite strong breastfeeding initiation rates, the number of infants exclusively breastfed below 6 months of age remains low at 41% worldwide [4]. For example in Australia, the breastfeeding initiation rate is 96%, yet only only 15% of Australian infants are exclusively breastfed at 6 months of age [5]. While women choose to stop breastfeeding for a multitude of reasons, a frequent motive to prematurely wean or introduce infant formula is inadequate milk supply [6, 7]. This is despite almost all women having the biological ability to produce adequate breastmilk for their infant [8]. Health professionals’ inability to provide expert advice and support to women as they establish breastfeeding has also been cited as a barrier to exclusive breastfeeding [8]. Inadequate support from midwives [9] and conflicting advice [10] are reported as adversely influencing breastfeeding outcomes. Recommendations to improve global breastfeeding rates include the provision of consistent, evidence based guidance and advice to all women [6, 7].

Midwives are uniquely placed to provide breastfeeding education and support to the woman and it is imperative that they have a sound understanding of the physiological underpinnings of breastfeeding. Supporting and guiding the woman in the initiation and establishment of breastfeeding is essential and includes supporting women to not only make sense of their infants feeding cues but also to understand how their breasts produce and sustain milk supply. Midwives initially learn about breastfeeding as students and continue to develop and build on this foundational knowledge throughout their studies, with the application of theory to practice in the clinical setting. However, the physiology of lactation is complex and midwifery students often struggle to grasp the concepts and make sense of the physiological underpinnings of the mammary gland and breastmilk production.

Current literature supports the need for midwifery tertiary education providers to be creative and offer a variety of strategies to support student learning, and the application of theory to practice including the theoretical underpinnings of breastfeeding [11]. Yet, a search by the lead author of educational repositories, including local and global texts and materials on breastfeeding physiology for midwifery students, revealed primarily content-dense and often complex reading resources. Other research has highlighted the value of using visual and audio mediums to support student teaching and learning, with animated instructional resources emerging as an important adjunct in this field [12–14]. A unique feature of animation is the ability to demonstrate change over time including, the dynamic physiological sequencing of events including interations and inflences [12]. The learner is able to visualize the changing states of events as they occur and therefore do not have to infer the changes, potentially reducing cognitive load.

There is however a paucity of support for the use of instructional animation resources in relation to midwifery education and in particular the complexities of breastfeeding physiology. This proof of concept study was undertaken to evaluate *Breastfeeding Hormones in Play* [15]*,* an instructional animation resource to teach breastfeeding physiology to student and practicising midwives and gain insights into the acceptability of this novel approach to learning.

This study describes the evaluation of *Breastfeeding Hormones in Play.* The objectives of the study were to: (i) explore the impact of the *Breastfeeding Hormones in Play* resource on participants’ understanding of the physiology of lactation; (ii) assess the extent to which *Breastfeeding Hormones in Play* stimulates participants to learn more about breastfeeding; (iii) evaluate the acceptability of *Breastfeeding Hormones in Play*; and (iv) evaluate the usability of *Breastfeeding Hormones in Play*.

### Development of the instructional animation resource

A team of academics with expertise in the science of breastfeeding, animation and film production developed the *Breastfeeding Hormones in Play* instructional animation resource. From written text, the concept was shaped using role-play, moving shapes on a whiteboard and finally imagining and creating engaging animated characters to tell the story of breastmilk physiology. Key elements that have been determined to optimise student engagement such as cueing and music [16] and the humanizing of characters [17] were incorporated into the design. The hormone characters came to life with personalities created by giving each a unique shape, colour and facial expression.

*Breastfeeding Hormones in Play* is an eight-minute instructional animation video produced using *Blender* software after creating and sourcing 3D models, set up simulations and particle emitters for the ‘flowing’ effect. The remainder of the animation was achieved with standard key framing. The video was edited using *Premiere Pro* and *After Effects* with music sourced from *Motion array* and *Getty Images* along with music specifically created for the animation by Deakin University. The video is freely available on YouTube.

## Methods

This proof of concept study employed a cross-sectional anonymous questionnaire to survey registered midwives (RM) and midwifery students regarding their experience of *Breastfeeding Hormones in Play*. The setting was online with midwifery students recruited from Deakin University and RM’s recruited from the Australian College of Midwives (ACM) membership. Snowball sampling was also employed to recruit midwives through professional networks of the research team. Selection criteria required that students had undertaken or be currently studying breastfeeding. Registered midwives in clinical practice were eligible to participate. Ethics approval for the study was obtained from Deakin University. Participant consent was implied by return of the anonymous survey.

Midwives and midwifery students were invited to participate via email in April 2018 (midwives) and September 2017 (students). The email contained a Participant Information Sheet and the link to the online questionnaire hosted on Qualtrics software; consent was implied by return of the online survey. The midwife survey included a link to *Breastfeeding Hormones in Play* video. Midwifery students had previously been exposed to the video within their undergraduate course.

The survey items were developed by the team, informed by a literature review and the objectives of the study and items were pretested with non-clinical midwives. The questionnaire for midwives and midwifery students had 28 and 21 items respectively. Most items contained a five point Likert scale of agreement, some professional details were collected and there was an open-ended question for additional comments. It was estimated the survey would take up to 10 min to complete. In view of the convenience sampling, a sample estimate was not calculated. Data was analysed using descriptive statistics including frequencies and percentages to summarise the data. Open-ended responses were analysed deductively using the objectives of the study to assist in coding.

## Results

One hundred and ten (*n* = 110) surveys from the two survey groups were received and analysed. One survey from each group was largely incomplete and removed, two student midwife responses from the registered midwife survey were also removed; a total of 81 midwife responses and 25 midwifery student responses were included in the analysis. Registered midwife demographics are presented in Table 1.

**Table 1:** Demographic characteristics of midwife participants

Characteristics	n	%
**Geographical employment setting**		
Metropolitan	33	40.7%
Regional	36	44.4%
Rural	10	12.3%
Remote	2	2.5%
Missing	2	2.5%
**Employment**		
Public Hospital	63	77.8%
Private Hospital	3	3.7%
Community setting	5	6.2%
Private midwifery practice	3	3.7%
General practice	0	0%
Obstetrician’s rooms	0	0%
Other, please specify	7	8.6%
**Area of work**		
Postnatal	17	21%
All areas	48	59.3%
Pregnancy Care	1	1.2%
Labour and Birthing	5	6.2%
Domiciliary	0	0%
Other, please specify	10	12.3%
**Average weekly work hours**		
1–8	10	12.3%
9–16	5	6.2%
17–24	17	21%
25–32	23	28.4%
33–40	19	23.5%
> 40	7	8.6%
**Attended Breastfeeding Professional Development in the last 12 months**		
No	34	41.5%
Yes	48	58.5%
**Current IBCLC**		
No	63	76.8%
Yes	15	18.3%
No, but have been in the past	4	4.9%

Over 72.3% (*n* = 73) of the RM participants practiced in the eastern states of Australia; Queensland, New South Wales and Victoria. All states and geographical employment settings were represented, however numbers of participants in other jurisdictions were small. South Australia and Western Australia were each represented with 3.7% (*n* = 3), Australian Capital Territory and the Northern Territory each were represented by 1.3% (*n* = 1), there were 2.5% (*n* = 2) of surveys returned with this demographic data missing. The majority of participants were employed across all areas of midwifery practice, in the public health system in either a metropolitan or regional centre. Most midwives worked more than 25 h per week (60.6%) and all participants practised in clinical settings that required use of breastfeeding knowledge. A small majority (58.5%) had engaged in breastfeeding professional development (PD) in the past 12 months, with metropolitan and remote midwives more likely to have attended than their regional or rural counterparts. Participants were asked if they held a current or lapsed International Board Certified Lactation Consultant (IBCLC) qualification as this qualification requires a heightened level of breastfeeding physiology knowledge compared to that of a Registered Midwife. The number of current or lapsed IBCLC in the sample was 23%, (*n* = 19).

### Impact of *Breastfeeding Hormones in Play* on midwives’ understanding of breastfeeding physiology

Registered midwives responded to questions related to knowledge and understanding of lactation physiology concepts such as terminology, hormone interplay and autocrine control (Table 2). Most midwives agreed or strongly agreed that the resource had a positive impact on their understanding of physiology concepts. On average, across items in Table 2, participants’ level of combined agreement was 82%, with more than half (55%) acknowledging that the resource had taught them something new about breastfeeding physiology. Participants who are currently, or have been IBCLC’s were more likely to disagree to acquiring new knowledge from the resource (52.6%) compared to those who had never held an IBCLC qualification (38%). Engaging with the resource also had an impact on midwives’ confidence about breastfeeding physiology with 65% agreeing their confidence levels were improved.

**Table 2:** Level of agreement of midwife participants to statements of the impact of *Breastfeeding Hormones in Play* on an understanding of breastfeeding physiology (*n =* 81)

Statement	Strongly agree n (%)	Agree n (%)	Neither agree or disagree n (%)	Disagree n (%)	Strongly disagree n (%)	Unsure n (%)
I learnt new knowledge by using the Breastfeeding Hormones in Play resource.	12 (14.8)	33 (40.7)	17 (20.9)	17 (20.9)	1 (1.2)	1 (1.2)
The Breastfeeding Hormones in Play resource has refreshed my understanding of breastfeeding physiology.	32 (39.5)	42 (51.8)	5 (6.1)	0	1 (1.2)	1 (1.2)
The Breastfeeding Hormones in Play resource refreshed my knowledge of the terminology of breastfeeding physiology.	20 (24.7)	40 (49.3)	14 (17.2)	2 (2.4)	4 (4.8)	1 (1.2)
The Breastfeeding Hormones in Play resource refreshed my knowledge of the interplay between breastfeeding hormones.	30 (37)	40 (49.3)	7 (8.6)	2 (2.4)	1 (1.2)	1 (1.2)
The Breastfeeding Hormones in Play resource refreshed my knowledge of the autocrine control of breastfeeding.	26 (32)	38 (46.9)	10 (12.3)	4 (4.82)	1 ( 1.2)	2 (2.4)
The Breastfeeding Hormones in Play resource helped me to feel more confident on the topic of breastfeeding physiology.	19 (23.4)	34 (41.9)	20 (24.6)	5 (6.1)	2 (2.4)	1 (1.2)

### Extent to which animation stimulated participants to learn more about breatfeeding (*n* = 81)

Of those participants who had not attended breastfeeding PD in the last 12 months, 47% indicated that they were motivated to continue PD on breastfeeding, with 6% disagreeing and the remainder (47%) were ambivalent. Of those who had attended breastfeeding PD in the last 12 months, 71% indicated that they were motivated to continue PD on breastfeeding and 4% disagreed. While overall the outcome for motivation to learn more about breastfeeding is favourable (60% agree/strongly agree) the outcome did not demonstrate an increased motivation in the majority of participants who had not completed any recent breastfeeding professional development.

### Acceptability of animation as a resource

The vast majority (96.3%) of participants indicated that they enjoyed watching the video and 96.3% found it complementary to other breastfeeding resources. Most (87.5%) agreed that the content was evidenced based. (Table 3).

**Table 3:** Level of midwives’ agreement to statements on acceptability of *Breastfeeding Hormones in Play* as a resource

Statement	Strongly agree n (%)	Agree n (%)	Neither agree or disagree n (%)	Disagree n (%)	Strongly disagree n (%)	Unsure n (%)
I enjoyed viewing the Breastfeeding Hormones in Play resource. (*n* = 80)	32 (40)	45 (56.3)	3 (3.7)	0	0	NA
The Breastfeeding Hormones in Play resource complements other breastfeeding resources. (*n* = 80)	31 (38.8)	46 (57.5)	3 (3.7)	0	0	NA
The Breastfeeding Hormones in Play resource is evidence based. (*n* = 80)	28 (35)	42 (52.5)	10 (12.5)	0	0	0
I would recommend the Breastfeeding Hormones in Play resource to other midwives. (*n* = 78)	32 (41)	39 (50)	6 (7.7)	1 (1.3)	0	NA
I would recommend Breastfeeding Hormones in Play resource to midwifery students. (*n* = 80)	50 (62.5)	27 (33.7)	0	1 (1.2)	2 (2.4)	NA

Participants’ willingness to discuss and share the resource also indicated acceptance of the *Breastfeeding Hormones in Play* as a learning resource. The majority of RM participants indicated they would recommend the resource to midwife colleagues (71%) and to midwifery students (96%). Qualitative comments from participants affirmed this instructional animation as an acceptable form of learning resource (*n* = 32). Comments including: *“I would love to share this resource as a teaching tool as this subject can be difficult to make interesting and easy to absorb”;* “*the animations help reinforce the explanations”* and “*the actions of the characters made their interaction and effect very clear”,* are representative of the responses. Participants also commented on the *“squigy sound effects”* and *“fun”* characteristics of the animation resource as positive features. It is clear from the qualitative comments that the participants engaged with the content presented as an instructional animation and that the effects used enhanced their experience.

A small number (*n* = 3) of participants indicated that the instructional animation was not their preferred modality of learning, for example one participant commented. *“I found the colored shapes a bit annoying”.* There were however, a number of suggestions provided to enhance the user experience of the animation. For example, another participant commented that having the name of the hormone characters displayed would make them easier to remember:“*I forgot which character was which so maybe if they had their names displayed the whole time?”*

### Useability of the resource

The useablity of the resource is reflected in both the quantitative and qualitative responses. Most 77.2% (*n* = 61) of the midwife respondants watched the video once, with the remainder watching more than once. Only one respondant had not watched the whole video.

There were a small number of participants (< 9%) who thought the video was too long or too short, the majority were satisfied with the length of the video.

Participants offered observations of the animation in regard to useability. Theming and analysis of qualitative data confirmed that the resource was ‘user friendly’ and the elements that had been included such as music, voice over, onscreen text, graphics and sound effects were well accepted and were reflected in participant comments about enjoyable aspects of the resource. In general, *Breastfeeding Hormones in Play* was viewed as a great resource for teaching and easy to follow. Representive quotes include: *“I think this is a great resource for visual learners”; “Great teaching resource, easy to watch and will be very useful for health professionals”; and “It was very easy to follow, although I did have prior knowledge it could very easily have been shown to people without this and they would have understood. Very user friendly”.* The few comments that offered an alternate view related to not liking the music and the duration being too long or too short.

### Midwifery students

The majority of midwifery student participants were in their second year of a four-year combined nursing and midwifery degree. All student participants had some exposure to postnatal care which included supporting women with breastfeeding, however, 44% had less than 160 h of clinical practice in this area. The majority of student participants (80%) had not sourced any additional breastfeeding education outside of their course. Student midwife characteristics are presented in Table 4.

**Table 4:** Characteristics of Student Midwife participants

Midwifery students’ characteristics	n	%
**Year of midwifery study**		
2nd year	11	44.0%
3rd year	5	20.0%
4th Year	8	32.0%
Post graduate	1	4.0%
**Completed breastfeeding education outside of course**		
No	20	80.0%
Yes	5	20.0%
**Shifts spent in postnatal setting (8 h duration)**		
Less than 6	0	0.0%
6–10	3	12.0%
11–20	8	32.0%
More than 20	14	56.0%

### Impact of animation on midwifery student participants understanding of breastfeeding physiology

The majority of midwifery students agreed or strongly agreed that the resource assisted them to understand the various facets of breastfeeding physiology, with combined agreement ranging 85.8–99% (Table 5). There was also a strong level of combined agreement that students were motivated to learn more about breastfeeding after viewing the *Breastfeeding Hormones in Play* resource.

**Table 5:** Impact on midwifery student participant understanding

Statement	Strongly agree ***n*** (%)	Agree n (%)	Neither agree or disagree n (%)	Disagree n (%)	Strongly disagree n (%)	Unsure n (%)
The *Breastfeeding Hormones in Play* resource has improved my understanding of breastfeeding physiology. (*n* = 24)	14 (58.3)	8 (33.3)	2 (8.3)	0	0	0
The *Breastfeeding Hormones in Play* resource helped me to understand the terminology of breastfeeding physiology.(*n* = 22)	11 (50)	9 (40.9)	2 (9)	0	0	0
The *Breastfeeding Hormones in Play* resource helped me to understand the interplay between breastfeeding hormones. (*n* = 22)	14 (63.6)	6 (27.4)	2 (9)	0	0	0
The *Breastfeeding Hormones in Play* helped me to understand the autocrine control of breastfeeding. (*n* = 21)	9 (42.9)	9 (42.9)	3 (14.2)	0	0	0

### Acceptability and usability of animation as a resource

Midwifery students responded most positively to statements on their attitudes to the *Breastfeeding Hormones in Play* resource with levels of combined agreement ≥95% (Table 6). Responding to a statement that they would recommend the resource to a friend, 75% strongly agreed.

**Table 6:** Acceptability and Useability of resource for midwifery students

Statement	Strongly agree n (%)	Agree n (%)	Neither agree or disagree n (%)	Disagree n (%)	Strongly disagree n (%)	Unsure n (%)
I enjoyed viewing the Breastfeeding Hormones in Play resource. (*n* = 21)	16 (79.2)	4 (19)	NA	0	0	NA
The Breastfeeding Hormones in Play resource complements other breastfeeding resources. (*n* = 21)	10 (47.6)	11 (52.4)	NA	0	0	NA
The Breastfeeding Hormones in Play resource is evidence based. (*n* = 21)	21 (52.4)	9 (42.8)	NA	0	0	1 (4.8)
I would recommend Breastfeeding Hormones in Play resource to midwifery students. (*n* = 20)	15 (75)	4 (20)	NA	1 (5)	0	NA

Over two thirds of students (68.4%) had discussed the instructional animation resource with other students. The number of times students viewed the resource varied with the majority viewing the animation three times or more (63.1%). The length of the resource suited most, with only 21% of participants judging it to be too short, 5% of students deeming the duration to be too long.

### Qualitative results from midwifery students

#### Impact on learning/ understanding

**Fig. 1:**
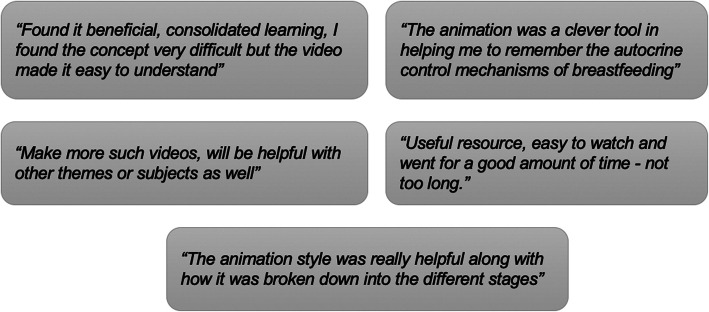
Examples of midwifery student responses of their experience using *Breastfeeding Hormones in Play*

Revealing their thoughts on their experience of using the instructional animation resource, the midwifery students commented on how the resource increased their understanding of breastfeeding physiology as illustrated in Fig. 1.

## Discussion

### Impact on learning/understanding

Breastfeeding physiology can be considered as a threshold concept. Land and Mayer [18] describe threshold concepts as a fundamental concept that requires a shift in perception that will facilitate the understanding of a difficult concept. These concepts are potential road blocks which prevent students progressing to the next level of learning and understanding. Angell and Taylor [19] support the notion of threshold concepts as a barrier to midwifery students when learning about breastfeeding and mammary gland physiology. Threshold concepts have been identified as troublesome for the student learner [18]. Within the context of this study, midwifery students expressed the conceptual difficulties of understanding the hormonal interactions underpinning breastfeeding physiology. For example, one student commented, *“reading about it is sometimes so complex”*. While for the experienced RM, knowledge is often tacit, this can have repurcussions if they don’t explicitly include physiology in their clinical interactions with students. As one student expressed, “*seeing the ‘surge’ of breastmilk due to oxytocin, which also cleared up for me why babies ‘suck, suck, suck, pause”.* The results of this study demonstrate that the *Breastfeeding Hormones in Play* resource enhanced midwifery students understanding of breastfeeding physiology. Khurshid et al. [20] reported similar findings, with 85% of optometry students in their study finding animation effective in assisting them to understand difficult concepts by presenting them in ways that they were able to easily understand. Similarly, Barak, Ashkar and Dori [21] found that students who had access to animation had a heightened understanding and better capacity to explain complex concepts.

Breastfeeding resources for RM’s are highly relevant and a requirement for ongoing professional development. Henderson and Redshaw [22] asserted that a woman’s breastfeeding success is influenced by the support and advice provided by the midwife. Further, the authors highlight that breastfeeding educational resources targeted specifically to midwives are integral to the delivery of reliable evidence based advice. Accessibility to breastfeeding PD has been found to be influenced by geographical location and population density. Edward et al. [23] suggests midwives working in rural and regional settings are less likely to have targeted breastfeeding PD compared to their metropolitan counterparts. Our study supports this finding, with results showing metropolitan and remote midwives were more likely to have attended breastfeeding PD than their regional or rural counterparts. The *Breastfeeding Hormones In Play* resource can make a meaningful contribution to the provision of breastfeeding PD, regardless of the RM’s geographical location.

Breastfeeding women are more likely to be accepting of advice and be able to contextualise it to their current breastfeeding experience when provided with a rationale [24]. In a study conducted by Swerts et al. [25] women identified that they were unsure about breastmilk production and this was at the forefront of many of their questions in the early postnatal period. It is imperative that both midwifery students and RM’s are confident in their knowledge about breastmilk production and are able to reassure and support women in their early breastfeeding experiences. This study has demonstrated that this instructional animation has the capacity to support midwifery students and RM’s in their understanding of the complex physiology of breastfeeding and translate this to clinical practice.

### Stimulation to learn more about breastfeeding

The results of this study supports the notion of an instructional animation resource inspiring midwifery students to want to further advance their knowledge and understanding of breastfeeding physiology. Evidence to support instructional animation as a motivator for students to further investigate the subject matter is sparce. Khurshid [20] found that use of animation as an approach to teaching undergraduate optometry students enhanced students interest in the subject matter. Similarly, Barak, Ashkar and Dori [21] found students were more motivated to continue to engage with science subjects when they were exposed to animated movies. This study contributes to this body of evidence, supporting the idea of concepts delivered as animation as a motivating factor by engaging students in the content.

### Acceptability of animation as a resource

Previous attitudes about the theory being dull and content dense were transformed with both groups reporting enjoyment engaging with the animation and 85% of student participants reporting that they had watched it multiple times. Paticipants in both groups acknowledged that this resource was aligned with other breastfeeding physiology learning material, which demonstrates they were able to easily link the information offered in the animation to other breastfeeding teaching artifacts. This was important, particularly for midwifery students, as the animation intention was not as a stand alone resource, but as complimentary to other resources. Participants’ endorsement of the resource as being evidence based, legitimises the information as being trustworthy and authentic and therefore unlikely to be a source of conflcting information.

Educators are well versed in the necessity to provide learning resources that cater to varied learning styles. Morris, Prankard and Lefroy [14] suggest that animation can engage a diverse range of learners by transforming abstract knowledge into images. The largely positive responses demonstrated that most found this style of learning suited their preferences, “*Great for visual learners”* (RM). However, the animation did not suit everyone as another RM commented, “*Too diagramatic and not helpful for me, sorry”.*

### Useability of the resource

**Fig. 2:**
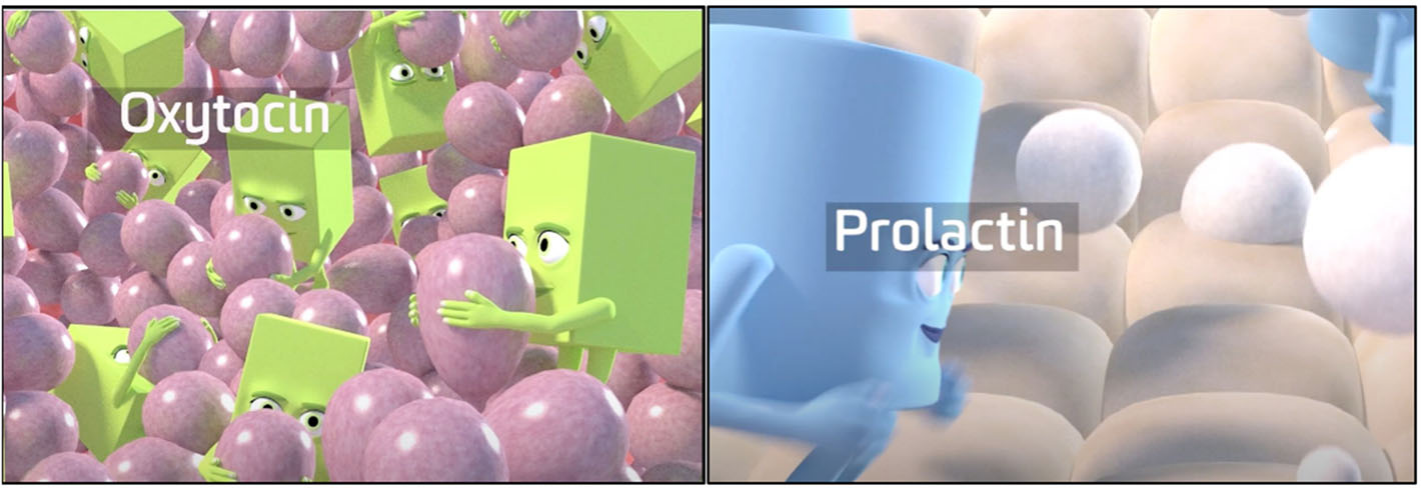
Labelling of hormones

The ability to visualise the changing state of the breast and how the interaction of the hormones create change, means the learner does not have to infer the changes [12]. This feature made animation an attractive option for learners. However, Kalyuga [26] suggests that continuous animations could be too cognitively demanding for novice learners and better suited to learners with previous knowledge of the content. Interestingly, qualitative feedback was similar between the two groups in this regard, with both reporting a struggle to recall the hormone characters. In response to this feedback additional cueing was incorporated into the animation by introducing more frequent labelling of hormones throughout as illustrated in Fig. 2.

There is a fine balance between engaging and distracting content. Brom et al. [17] found that the learners state of engagement influences the cognitive resources that they will devote to the content. Participants in this study did not articulate any significant issues with distraction, apart from some isolated comments from some of the RM’s: “*The background music is too relaxing! It made me sleepy*”; *“I was distracted a little from listening to the narrator as I was watching the ‘story’ being told by the 3D shapes”.* Acknowledging this feedback, the production team reviewed and made adjustments to some of the background music. Overall, the feedback reflected a well balanced engaging resource. Lucas and Abd Rahim [16] found elements such as: cartoon visual style, visual cues on essential information, abstract images, short duration (less than 12 min), human voice, conversational, sound effect (music), verbal narration with visuals and minimal onscreen text, to all be elements of popular YouTube animations. Each of these elements are present in *Breastfeeding Hormones in Play,* together with background music and humour. These were well received by those evaluating the resource, articulating the most enjoyable aspects of the animation as: “*The use of cartoon characters” (midwifery student)*; *“the squigy sound effects”; “short duration, clear dialogue”; “the clear labelling was great”* and *“interesting, funny and clear graphics” (RM’s).*

Overall, respondants were positive about the useability of this resource. The production team have been responsive to the feedback and have identified capacity for enhancement and modification of the animation to further improve the learning experience. The team, committed to continual improvement of the resource, reviewed and altered music tracks, included additional cueing and included an enhanced introduction and conclusion to the video. The video has also been divided up into short, single concepts that are annotated with knowledge testing questions. This strategy encourages active learning and provides informal formative feedback for midwifery students. In the first 18 months since publishing the *Breastfeeding Hormones In Play* on YouTube there had been over 8000 views.

### Strengths

Evaluation of innovative educational resources like *Breastfeeding Hormones in Play* are of critical importance and afford the opportunity to respond to the end-user and improve the resource. Underpinned by a commitment to improve the breastfeeding support, education and care of women, surveying both midwifery students and midwives is a strength of this study.

### Limitations

This study was limited by the small sample and a cross-sectional descriptive survey design. In particular, rural and remote midwives were not well represented in comparison to metropolitan and regional midwives. While there was strong support for the instructional animation resource in this study, caution needs to be taken to not extrapolate these results to a broader audience.

## Conclusions

The complex physiology of lactation and the mammary gland has been presented as a fun and engaging resource. The *Breastfeeding Hormones In Play* instructional animation resource takes the learner on a journey into the inner workings of the female body via clear and effective audio and visual communication. This instructional animation resource has enabled the learner to make the connection between theory and practice and has provided the tools and language with which to explain these concepts to the woman. Although not the original intention, this instructional animation resource has also proved to be a useful tool for RM’s who wish to update their understanding of breastfeeding physiology. The findings from this proof of concept study support the pedagogical advantages of instructional animation. Further, educators should be encouraged and feel confident to consider animation technology as both an engaging and effective teaching resource.

## Data Availability

The datasets used and/or analysed during the current study are available from the corresponding author on reasonable request.

## Abbreviations

3D: Three dimensional
IBCLC: International Board Certified Lactation Consultant
PD: Professional development
RM: Registered Midwife
